# Evolutionary Game Analysis of the Social Co-governance of E-Commerce Intellectual Property Protection

**DOI:** 10.3389/fpsyg.2022.832743

**Published:** 2022-02-18

**Authors:** Ji Li, Chunming Xu, Lufei Huang

**Affiliations:** ^1^School of Management, Shanghai University, Shanghai, China; ^2^Shanghai International College of Intellectual Property, Tongji University, Shanghai, China; ^3^School of Fintech, Shanghai Lixin University of Accounting and Finance, Shanghai, China

**Keywords:** e-commerce, social co-governance, intellectual property protection, evolutionary game, rights holder

## Abstract

By introducing the theory of social co-governance into the field of e-commerce intellectual property protection, this paper builds an evolutionary game model among the government, e-commerce platforms, and rights holders, and studies the conditions under the stakeholders form a stable equilibrium state under different constraints. Combined with numerical simulation, the influence of individual factors and factor combinations on the system stability is analyzed. Results shows that: Strictly controlling the action costs and response costs of all parties can enhance their willingness to actively deal with infringement issues; reasonable adjustment of the reward and punishment measures of government supervisory agencies can produce sufficient reverse shock and positive guidance to platform and operators; penalties should be imposed on government supervisory agencies that are not sufficiently supervised; strengthen the construction of the social environment for intellectual property protection, improve the social benefits of actively responding to infringement issues, and increase the sense of acquisition by the government, platforms and rights holders. And it provides certain positive references and suggestions for the government to formulate relevant policies.

## Introduction

In China, where the economy and social system are constantly evolving and changing, the internet economy, especially e-commerce business activities, has ushered in a new era of development (Kwak et al., 2019). According to relevant statistics, the scale of Chinese internet users has reached 989 million, of which online shopping users account for ~80%. According to the *China E-Commerce Report 2020* issued by the Department of E-commerce and Information Technology of the Ministry of Commerce of China, China's national e-commerce transaction volume in 2020 reached 37.21 trillion yuan, of which the national online retail sales reached 11.76 trillion yuan, ranking first in the world for eight consecutive years. This not only reflects the extremely high commercial value and huge social influence of e-commerce but also confirms that e-commerce has ushered in a new era in China (Huang and Li, 2019). In the post-epidemic era, the e-commerce market is ushering in structural reforms while influencing and changing consumers' consumption habits (Beckers et al., 2021).

However, at the same time, the problems associated with making and selling counterfeit products and infringing upon intellectual property rights in the field of e-commerce have gradually become prominent and caused many disputes (Mudrytska, 2020). Against the background of trade globalization, intellectual property has emerged as the key issue of global innovation policy (Archibugi and Filippetti, 2010). At present, China's intellectual property protection system is supervised by many parties (Brander et al., 2017), so China should improve the level of intellectual property protection. According to *China E-commerce User Experience and Complaint Monitoring Report*, thanks to China's emphasis on intellectual property protection and the continuous promulgation of relevant laws, regulations and policies in recent years, the number of infringement complaints received in China's e-commerce sector in 2020 was 45.59% lower year-on-year, but the total number of complaints has remained high. The investigation report of the High People's Court of Zhejiang Province pointed out that from 2014 to 2018, there were more than 3,000 civil cases of first instance involving the intellectual property rights of e-commerce platforms in Zhejiang Province each year. These cases illustrate the rapid increase in the number of annual cases, the range of case types covering multiple intellectual property fields, and the uneven geographical distribution of cases.

Intellectual property infringements are frequently industry-specific and involve strong professional knowledge. Government supervisory agencies and e-commerce platforms often have incomplete defense of intellectual property rights. In the presence of loopholes in the protection of intellectual property rights in the digital age, we usually apply legal remedies to provide protection and technical protection measures to strengthen the level of protection. However, technological protection measures can be avoided by circumvention techniques or brute force (Lucchi, 2005). Therefore, we need to find innovative ways to protect intellectual property rights to increase the effectiveness of protection in the e-commerce field (Deng et al., 2019; Lazariuc, 2021; Lazariuc and Lozovanu, 2021). The National People's Congress of China deliberated and passed the *E-Commerce Law* in 2018 and began to implement it in 2019. It regulates many issues of social concern. However, the inability of the law to keep pace with digital advancements is compounded by outdated rationales and traditional practices (Wu et al., 2021), and new issues keep emerging (Ge and Chen, 2021). In addition, the responsibility for protecting intellectual property rights undertaken by e-commerce platforms is too great, and the participation of other entities is limited, which has also led to inadequate supervision (Damanpour and Damanpour, 2001). Therefore, the Chinese government proposed for the first time in the *Report of the Work of the Government* in 2014 that the theory of social co-governance should be introduced into China's social governance work. In 2019, the policy document *Opinions on Strengthening the Protection of Intellectual Property Rights* pointed out that it is necessary to strengthen social supervision and co-governance of intellectual property rights through the establishment and improvement of the social co-governance model. The introduction of the theory of social co-governance into China's intellectual property protection field is a creative new measure.

As a victim of intellectual property infringement, intellectual property rights holders usually possess certain industry information resources as well as professional capabilities, which can provide effective help for the governance of infringements (Grzegorczyk, 2020). By cooperating with government departments and e-commerce platforms, intellectual property rights holders can assist in the prevention and guide the control of infringements, and they can supervise and restrict infringement control activities. In recent years, an increasing number of rights holders have hoped to take intellectual property rights in their own hands, seek remedies outside the legal system, and exercise intellectual property self-service through a series of related methods (Adler and Fromer, 2019). At present, some platforms in China have begun to try to invite rights holders to participate in the protection of intellectual property rights. For example, Alibaba's IPP platform has 524 rights holders settled from August 2019 to June 2020, which is nearly double the number in 2018. However, this is just one platform attempt, and there are still many problems that need follow-up research to solve.

With reference to the existing literature, there are very few related studies on the construction of e-commerce intellectual property protection systems under the theory of social co-governance based on evolutionary games. Therefore, the main purpose of this paper is to build a tripartite evolutionary game model of the government, e-commerce platforms, and rights holders, analyze the relationship between them, reveal the strategy of the evolution mechanism, explore the construction methods of e-commerce intellectual property protection systems, and finally, provide suggestions for the construction of China's e-commerce intellectual property social co-governance system and strengthen China's e-commerce intellectual property protection. Based on the research purpose and existing literature, the main problems to be solved in this paper are as follows:

1. With the introduction of rights holders in the social co-governance system, what factors affect the decision-making of stakeholders?
2. How can the strategic stability of each stakeholder be adjusted?
3. How can we guide and build a more stable e-commerce intellectual property social co-governance system?

In response to the above problems, this paper adopts the evolutionary game analysis method to construct a tripartite evolutionary game model of the government, e-commerce platform and rights holders and analyses the stability of each party's strategy and the influence of each element on the tripartite strategy choice.

At the same time, this paper discusses the influence of individual factors and factor combinations in the replication dynamic system on the stability of the system through a numerical simulation with MATLAB software and verifies the validity of the model under different initial conditions. Finally, this paper puts forward suggestions for the construction of China's e-commerce intellectual property social co-governance system.

The rest of the paper is organized as follows. Section Literature Review reviews the literature related to e-commerce intellectual property protection, social co-governance and evolutionary game theory. Section Social Co-governance Evolutionary Game Model establishes a stakeholder model based on model assumptions and defines relevant parameters. Section The Evolutionary Game Model Solution and Analysis evaluates the stability of the model and obtains some analysis results. Section Simulation Analysis describes the results of evolutionary game simulation and verifies the validity of the model. Section Suggestions and Conclusions summarizes and analyses stakeholders and models and gives policy suggestions and conclusions.

## Literature Review

### Mechanism of E-Commerce Intellectual Property Infringement

In recent years, technological progress and rapid development on the internet have increased enthusiasm for economic activities, and its popularity has significantly changed the traditional system of human interaction and communication (Brocke et al., 2018). The openness of the internet has doubled the breadth and depth of information dissemination, and e-commerce activities have also become closely related to our daily lives in this global revolution. The low cost, high flexibility, and global nature of e-commerce activities make the development of e-commerce particularly rapid (Babenko et al., 2019; Rathnayake, 2021; Salehi et al., 2021). However, the inherent information asymmetry characteristics of e-commerce can make it difficult for buyers and sellers to transmit product quality information (Lee et al., 2005; Christozov et al., 2009; Hossain et al., 2021; Lu and Chen, 2021), and the buyer's quality assessment of the sellers cannot be passed on to subsequent buyers (Pan, 2011; Devos et al., 2012). This will make high-quality sellers unable to obtain sufficient market recognition, resulting in the “lemon effect” in the e-commerce market (Hossain et al., 2018, 2021), which is particularly difficult in the context of the continuous change of new information technology and software applications (Babenko et al., 2019). Information asymmetry in the field of e-commerce has become a very serious problem (Gregg and Scott, 2006; Wei and Ho, 2019). Eliminating the asymmetry of information has become the key to reducing the lemon effect in the e-commerce market (Lee et al., 2005; Pavlou, 2005; Pavlou et al., 2007; Bove and Benoit, 2020; Dang et al., 2020).

The uncertain and fictitious nature of this kind of information has also led to the frequent occurrence of intellectual property rights infringements on the internet, which has had unprecedented impacts on the protection of rights holders. At present, intellectual property infringement issues of e-commerce platforms mainly target the areas of patents, trademarks and copyrights (Ma, 2020). Among the abovementioned infringements, when dealing with related issues, the fixation of evidence, preservation of the most evidence and their assessment are difficult (Hunter et al., 2021).

In addition, the basic literacy and moral awareness of internet users regarding intellectual property are uneven, and the awareness of respecting intellectual property rights throughout the whole society is not perfect (Lehman, 2006; Sun et al., 2021). Only by improving relevant systems and strengthening network management can a social environment conducive to good protection of intellectual property rights be formed (Wang, 2020, 2021). Xu and Qi (2017) also pointed out that e-commerce transactions have a certain degree of dependence on internet resources, while the development of the internet in China is not balanced, and the digital divide is obvious, which also leads to the inevitable infringement of e-commerce intellectual property rights.

### The Application and Practice of Social Co-governance Theory

With the continuous changes and developments of the global economic system, the government's original supervisory capabilities have not been adapted to social development (Offe, 2018), and it is difficult to coordinate these capabilities with the growing management demands of social entities (Wang and Lan, 2017). Therefore, adjusting the social supervision system and coordinating government supervisory agencies and social subjects to jointly manage social issues jointly promoted the development of social co-governance theory (Herod et al., 1998). Social co-governance is a model of social autonomy under government supervision that is different from governance and social autonomy without government supervision (Coglianese and Lazer, 2003; Baur et al., 2017; Hong et al., 2020). Over the past 30 years of China's reform and opening-up, society has undergone revolutionary changes, and economic and industrial structural changes have also brought many problems. The current governance model has been unable to meet China's needs, and the co-governance of multiple entities has become the only approach for China's development (Wang and Wang, 2017; Liu, 2018; Song, 2020). At present, the theory of social co-governance is applied mainly in the fields of food safety risk management and environmental protection (Offe, 2018). Kang (2019) discussed the changes and continuation of China's food safety governance issues. Wu et al. (2018) proposed the connotation and operational logic of food safety co-governance and proposed a prospect for future research on food safety risk co-governance. Yang and Li (2021) put forward policy suggestions for establishing a behavior integral system and information platform for the social co-governance of ecological and environmental protection. The introduction of the concept of social co-governance in the protection of e-commerce intellectual property rights is an innovative approach, and there are very few studies in this field. Kleinwächter (2006) pointed out that internet co-governance activities have developed into a system involving multi-stakeholder participation that requires consultation, coordination, and cooperation. Marsden (2008) wrote that internet governance must respect the social and economic rights and responsibilities of consumers and described the emerging agenda of “multi-stakeholder governance”. To study a social co-governance system for e-commerce intellectual property rights protection is to build a supervision system involving the government, e-commerce, industry associations, media, and the public to create a “big protection” pattern of intellectual property rights. Liu et al. (2018) proposed encouraging social organizations such as e-commerce business circles, industry associations, non-profit organizations, third-party evaluation agencies and other social organizations to participate in e-commerce intellectual property protection and social governance. At present, there are few studies on how to coordinate multiple stakeholders and build a complete social governance system for e-commerce intellectual property protection. Therefore, analyzing and discussing the social co-governance model of e-commerce intellectual property protection with the participation of rights holders will provide a useful supplement and reference for existing research and practical work.

### Application of Evolutionary Game Theory in Intellectual Property Protection

The evolutionary game reflects the continuous learning and improvement process of the players in the game and can effectively demonstrate the evolution of their learning mechanism and strategy (Weibull, 1997; Xia et al., 2018; Fan et al., 2021). In recent years, scholars in the field of intellectual property protection have gradually conducted more analysis and research using evolutionary game theory. Based on evolutionary game theory, Yang et al. (2018) analyzed government-industry-university-research intellectual property cooperation behavior and influencing factors from market and administrative supervision mechanisms. Although there are few studies on the social co-governance of intellectual property based on the evolutionary game method, evolutionary game analysis in the field of food safety social co-governance has mature research for our reference (Shen and Wei, 2020; Song et al., 2020). Scholars have also put forward opinions and suggestions of practical significance for building a food safety social co-governance system. Therefore, it is feasible to use the evolutionary game analysis method to study the intellectual property protection of e-commerce platforms from the perspective of social co-governance. In addition, many scholars have introduced social co-governance theory in e-commerce-related research. Li et al. (2018) constructed an evolutionary game model of privacy protection between enterprises and consumers based on the personalization of e-commerce and obtained a win-win result. This further affirms the feasibility of evolutionary game theory in the research of e-commerce intellectual property protection.

## Social Co-governance Evolutionary Game Model

### Model Description

The introduction of social co-governance theory into China's e-commerce activities is innovative. It is generally believed that in an evolutionary game system, participants are bounded rational. Stakeholders adjust their strategies by observing the decisions of others and comparing their own benefits. Therefore, studying the dynamic evolution process of stakeholders' strategies in the e-commerce intellectual property protection social co-governance system can better help us build a social co-governance system that suits the actual development of Chinese society. This paper constructs a game model for stakeholders, including the government, e-commerce platforms and rights holders, and studies the problems existing in the construction of the social co-governance system.

Government supervision departments are the main agency responsible for intellectual property protection and infringement handling in China. They are the key departments for policy formulation and administrative law enforcement. The government can effectively regulate the protection of e-commerce intellectual property rights by setting relevant measures in line with reality. However, how to carry out more detailed e-commerce intellectual property protection is a key issue that the Chinese government needs to consider. In China, against the background of increasing social attention to intellectual property rights, more effective protection and supervision actions can guarantee the government's credibility. That is, once there is an unfavorable problem of intellectual property protection, it is likely to reduce the credibility of relevant institutions.

E-commerce platforms are online service providers that provide transaction platforms for e-commerce entities. Therefore, they are responsible for guiding and managing the intellectual property activities that occur on the platform. At present, in the intellectual property protection work of China's e-commerce platforms, there are still problems such as high costs and unclear definition of pre-regulatory responsibilities. The E-Commerce Law and related laws and regulations are not yet complete. This will increase the possibility of platforms choosing to negatively respond to infringement issues to maximize their own interests, which is irresponsible to legal operators, rights holders, and consumers. Therefore, it is worth studying how to improve enthusiasm for intellectual property protection of e-commerce platforms and formulate reasonable guidance and management measures.

The holders of intellectual property rights will be directly affected when an infringement event occurs, which may cause them to incur irreparable losses. At present, an increasing number of rights holders are willing to actively participate in intellectual property protection activities. However, problems such as the poor connection between rights holders and government supervision departments or e-commerce platforms and the unapparent benefits of rights protection also affect the willingness of rights holders to take the initiative to protect themselves.

In addition, based on China's gradually improving social environment that respects intellectual property rights, the public's attention to stakeholders will also affect the social benefits of all parties and thus, the behavior of stakeholders.

### Model Assumptions and Parameters

In the e-commerce intellectual property social co-governance system with the participation of rights holders, stakeholders will comprehensively consider various benefits and make optimal decisions after repeated gaming, evolution and mutual learning activities. According to the model description and the specific requirements of intellectual property protection, we propose the following hypotheses:

1. We assume that the strategies of the government are (strict supervision, loose supervision), that is, the degree of looseness and strictness of the supervision and punishment mechanism. The probability that the government chooses strict supervision is represented as *x*, and the probability of loose supervision is represented as 1 − *x*. Similarly, the strategies of the e-commerce platforms are (active response, passive response), that is, how actively the e-commerce platform deals with intellectual property infringement issues that occur within the platform. *y* represents the probability of e-commerce platforms actively responding infringement issues. Correspondingly, 1 − *y* represents the probability of e-commerce platforms passively responding to infringement issues. In addition, we assume that the strategies of rights holders are (active rights protection, passive rights protection), that is, rights holders act in advance in the face of possible infringement issues, take the initiative to attack in the face of infringement issues that have already occurred, or choose to defend rights due to losses after infringement issues occur. The rights holders will cause different infringement losses and gains due to different strategic choices. The probabilities of the rights holder's active and passive protection are *z* and 1 − *z*, respectively.
2. E-commerce platforms adopting different management attitudes will incur corresponding costs. The cost of e-commerce platforms actively responding to infringement issues on the platform is *C*_*pa*_, and the social benefits for e-commerce platforms are currently *S*_*p*_. When an e-commerce platform passively responds to infringement issues, the cost is *C*_*pp*_, and the social benefits are *L*_*p*_. According to the actual situation, it is not difficult to obtain *C*_*pa*_ > *C*_*pp*_ and *S*_*p*_ > *L*_*p*_.
3. Government supervision departments carrying out different intensities of supervisory activities will incur corresponding costs. Under different intensities, government supervision departments can reward and punish platforms according to their different attitudes toward dealing with related issues and can impose fines on platform operators who infringe on intellectual property rights. When government supervision departments choose strict supervision, the supervision cost and social benefits are *C*_*g*_ and *S*_*g*_, respectively, and the government supervision departments will fine *P*_*s*_ for the infringement of platform operators. At this time, if the e-commerce platform actively responds to infringement issues, the government will reward the platform with *M*_*p*_, and if the e-commerce platform passively responds to infringement issues, the government will impose a fine *P*_*p*_ on the platform. When the government supervision departments choose loose supervision, it is impossible to obtain the information on the strategy choice of the e-commerce platforms and the platform operators, and the government supervision department does not provide rewards and punishments. At this time, the social benefits obtained by the government supervision departments are *L*_*g*_, where *L*_*g*_ < *S*_*g*_; if the e-commerce platforms passively respond to infringement issues, which leads to the disadvantage of intellectual property protection, the government supervision departments will be held accountable by the superior authority, and the administrative penalty will be *P*_*g*_.
4. The cost for the rights holders to prevent and take the initiative to protect their legitimate rights and interests in advance is *C*_*ha*_, and the rights holders' loss is *G*_*ha*_; at this time, the docking costs between the rights holders and the e-commerce platforms that passively respond to infringement issues is *C*_*ph*_ (the docking costs can be ignored when the e-commerce platforms actively respond to infringement issues); the government supervision departments that choose strict supervision have response costs of *C*_*gh*_ (no response costs under loose supervision) for the rights holders to actively protect their rights, and the rights holders have corresponding costs savings. The cost of passive rights protection, after the rights holders learn of the infringement, is *C*_*hp*_ (*C*_*ha*_ > *C*_*hp*_), and the rights holders' loss is *G*_*hp*_ at this time.

**Figure 1:**
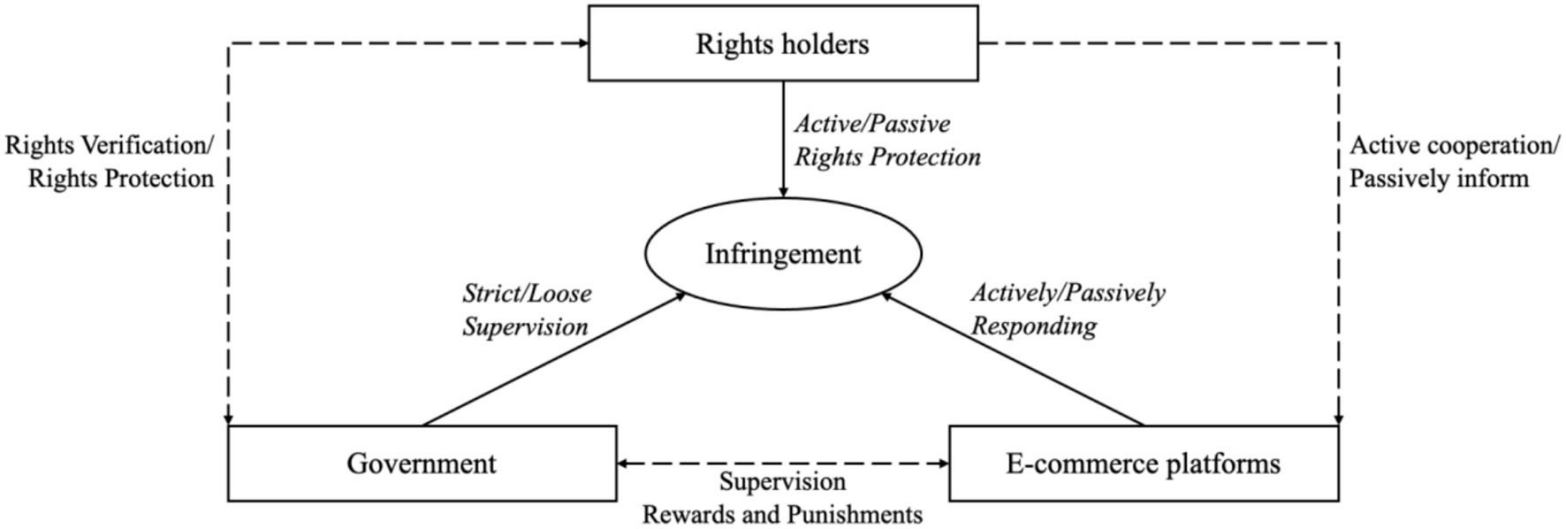
System diagram of e-commerce intellectual property social co-governance.

The relationship among the government, e-commerce platforms, and rights holders in this paper is shown in Figure 1. The corresponding parameters are described in Table 1.

**Table 1:** Parameter descriptions.

**Stakeholder**	**Parameter**	**Description**
The government	*x*	The probability that the government chooses strict supervision
	*C*_*g*_	The cost of strict supervision for the government
	*P*_*s*_	The fines for infringements of platform operators under strict government supervision
	*P*_*g*_	The superior authority's penalties for the government when the government chooses loose supervision, and the e-commerce platforms passively responds to infringement issues that are disadvantageous to intellectual property protection
	*S*_*g*_	The social benefits of the government's strict supervision
	*L*_*g*_	The social benefits of the government's loose supervision
E-commerce platform	*y*	The probability that e-commerce platforms choose to actively respond
	*C*_*pa*_	The cost of the e-commerce platforms actively responding to the infringement issues
	*C*_*pp*_	The cost of the e-commerce platforms passively responding to the infringement issues
	*M*_*p*_	The rewards for e-commerce platforms that actively respond to infringement issues when the government chooses strict supervision
	*P*_*p*_	The fines imposed on e-commerce platforms that passively respond to infringement issues when the government chooses strict supervision
	*S*_*p*_	The social benefits of the e-commerce platforms' active response
	*L*_*p*_	The social benefits of the e-commerce platforms' passive response
Rights holders	*z*	The probability that rights holders choose active rights protection
	*C*_*ha*_	The cost of active rights protection for rights holders
	*C*_*hp*_	The cost of passive rights protection for rights holders
	*C*_*ph*_	The cost of docking between the two when rights holders actively protect their rights and e-commerce platforms passively respond to infringement issues
	*C*_*gh*_	The response cost of the government when rights holders actively protect their rights and the government choose strict supervision
	*G*_*ha*_	The loss when rights holders take the initiative to defend their rights
	*G*_*hp*_	The loss when rights holders passively defend their rights

### Model Designing

According to the above assumptions, the evolutionary game model payoff matrix among the government, e-commerce platforms, and rights holders can be obtained as shown in Table 2.

**Table 2:** Payoff matrix among the government, e-commerce platforms, and rights holders.

**Government**	**E-commerce platform**	**Rights holders**	
		**Active rights protection (z)**	**Passive rights protection (1 − z)**
Strict supervision (*x*)	Active response (*y*)	(−*C*_*g*_ − *C*_*gh*_ − *M*_*p*_ + *P*_*s*_ + *S*_*g*_, − *C*_*pa*_ + *M*_*p*_ + *S*_*p*_, − *C*_*ha*_ + *C*_*gh*_ − *G*_*ha*_)	(− *C*_*g*_ − *M*_*p*_ + *P*_*s*_ + *S*_*g*_, − *C*_*pa*_ + *M*_*p*_ + *S*_*p*_, − *C*_*hp*_ − *G*_*hp*_)
	Passive response (1− *y*)	(− *C*_*g*_ − *C*_*gh*_ + *P*_*p*_ + *P*_*s*_ + *S*_*g*_, *C*_*pp*_ − *C*_*ph*_ − *P*_*p*_ + *L*_*p*_, − *C*_*ha*_ − *C*_*ph*_ + *C*_*gh*_ − *G*_*ha*_)	(− *C*_*g*_ + *P*_*p*_ + *P*_*s*_ + *S*_*g*_, − *C*_*pp*_ − *P*_*p*_ + *L*_*p*_, − *C*_*hp*_ − *G*_*hp*_)
Loose supervision (1− *x*)	Active response (*y*)	(*L*_*g*_, − *C*_*pa*_ + *S*_*p*_, − *C*_*ha*_ − *G*_*ha*_)	(*L*_*g*_, − *C*_*pa*_ + *S*_*p*_, − *C*_*hp*_ − *G*_*hp*_)
	Passive response (1− *y*)	(− *P*_*g*_ + *L*_*g*_, − *C*_*pp*_ − *C*_*ph*_ + *L*_*p*_, − *C*_*ha*_ − *C*_*ph*_ − *G*_*ha*_)	(− *P*_*g*_ + *L*_*g*_, − *C*_*pp*_ + *L*_*p*_, − *C*_*hp*_ − *G*_*hp*_)

In this paper, *U*_*ij*_ represents the expected benefits of the *i* stakeholder under the *j* strategy, where *i* = *g*, *p*, and *h* represent the three stakeholders of the government, e-commerce platforms, and rights holders, respectively. *j* = 1, 2, and 0 represent the first strategy, the second strategy and the average expected benefits, respectively.

Therefore, the expected benefits of strict supervision and loose supervision by the government are *U*_*g*1_ and *U*_*g*2_, respectively. According to the above payoff matrix, the expected returns of the two different strategies can be calculated as follows:

(1) $$\begin{array}{l} U_{g1}=-y\left(M_{p}\;+\;P_{p}\right)\;-\;zC_{gh}\;-\;C_{g}\;+\;P_{p}\;+\;P_{s}\;+\;S_{g}\\ U_{g2}=yP_{g}\;-\;P_{g}\;+\;L_{g} \end{array}$$

The average expected earnings of government can be calculated as:

(2) $$\begin{array}{l} U_{g0}\;=\;xU_{g1}\;+\;\left(1\;-\;x\right)U_{g2} \end{array}$$

The replication dynamics equation for government can be achieved as follows:

(3) $$\begin{array}{l} F\left(x\right)\;=\;\frac{dx}{dt}\;=\;x\left(x\;-\;1\right)[y\left(M_{p}\;+\;P_{p}\;+\;P_{g}\right)\\ \;+zC_{gh}\;+\;\left(C_{g}\;-\;P_{p}\;-\;P_{s}\;-\;P_{g}\;-\;S_{g}\;+\;L_{g}\right)] \end{array}$$

Similarly, the expected benefits of the e-commerce platform that actively and passively respond to infringement issues are *U*_*p*1_ and *U*_*p*2_, which can be calculated as:

(4) $$\begin{array}{l} U_{p1}=xM_{p}\;-\;C_{pa}\;+\;S_{p}\\ U_{p2}=-xP_{p}\;-\;zC_{ph}\;-\;C_{pp}\;+\;L_{p} \end{array}$$

The average expected earnings of e-commerce platforms can be calculated as:

(5) $$\begin{array}{l} U_{p0}=yU_{p1}\;+\;\left(1\;-\;y\right)U_{p2} \end{array}$$

The replication dynamics equation for e-commerce platforms can be achieved as follows:

(6) $$\begin{array}{l} F\left(y\right)=\frac{dy}{dt}=y\left(y\;-\;1\right)[-x\left(M_{p}\;+\;P_{p}\right)\;-\;zC_{oc}\\ \;+\left(C_{pa}\;-\;C_{pp}\;-\;S_{p}\;+\;L_{p}\right)] \end{array}$$

The expected benefits of the rights holders' active rights protection and passive rights protection are *U*_*h*1_ and *U*_*h*2_, respectively, which can be calculated as:

(7) $$\begin{array}{l} U_{h1}=xC_{gh}\;+\;yC_{ph}\;-\;C_{ha}\;-\;C_{ph}\;-\;G_{ha}\\ U_{h2}=-C_{hp}\;-\;G_{hp} \end{array}$$

The average expected earnings of rights holders can be calculated as:

(8) $$\begin{array}{l} U_{h0}=zU_{h1}\;+\;\left(1\;-\;z\right)U_{h2} \end{array}$$

The replication dynamics equation for rights holders can be achieved as follows:

(9) $$\begin{array}{l} F\left(z\right)=\frac{dz}{dt}=z\left(z\;-\;1\right)[-xC_{gh}\;-\;yC_{ph}\;+\;(C_{ha}\;-\;C_{hp}\\ \;+C_{ph}\;+\;G_{ha}\;-\;G_{hp})] \end{array}$$

## The Evolutionary Game Model Solution and Analysis

According to Formulas (3), (6), and (9), we construct the functions as follows:

(10) $$\begin{array}{l} G\left(y\right)=y\left(M_{p}\;+\;P_{p}\;+\;P_{g}\right)\;+\;zC_{gh}\;\\ \;+\left(C_{g}\;-\;P_{p}\;-\;P_{s}-P_{g}\;-\;S_{g}\;+\;L_{g}\right),\\ H\left(x\right)=-x\left(M_{p}+P_{p}\right)-zC_{ph}+\left(C_{pa}-C_{pp}\;-\;S_{p}\;+\;L_{p}\right),\\ J\left(y\right)=-xC_{gh}\;-\;yC_{ph}\;+\;\left(C_{ha}\;-\;C_{hp}\;+\;C_{ph}+G_{ha}-G_{hp}\right) \end{array}$$

When *G*(*y*) = 0, *H*(*x*) = 0, *J*(*yx*) = 0, there are

(11) $$\begin{array}{l} y^{*}=\frac{-C_{g}\;+\;P_{p}\;+\;P_{s}\;+\;P_{g}\;+\;S_{g}\;-\;L_{g}\;-\;zC_{gh}}{M_{p}\;+\;P_{p}\;+\;P_{g}},\\ x^{*}=\frac{C_{pa}\;-\;C_{pp}\;-\;S_{p}\;+\;L_{p}\;-\;zC_{ph}}{M_{p}\;+\;P_{p}},\\ y^{**}=\frac{C_{ha}\;-\;C_{hp}\;+\;C_{ph}\;+\;G_{ha}\;-\;G_{hp}\;-\;xC_{gh}}{C_{ph}} \end{array}$$

### Analysis of Individual Strategy Stability

With reference to the properties of evolutionarily stable strategies and differential equations, if *F*(*x*) = 0 and $\frac{dF\left(x\right)}{dx}\;<\;0$, then the probability of e-commerce platforms choosing regulation is in a stable state. *F*(*y*) and *F*(*z*) are similar. Based on this, we can analyze the strategy stability of the government, e-commerce platforms, and rights holders.

#### Analysis of Strategy Stability of the Government

Proposition 1.

1. When *y* = y^*^, all strategies are in a stable state.
2. When *y* ≠ y^*^, *F*(*x*) = 0, *x* = 0 and *x* = 1 are the stable points of *x*.

**Figure 2:**
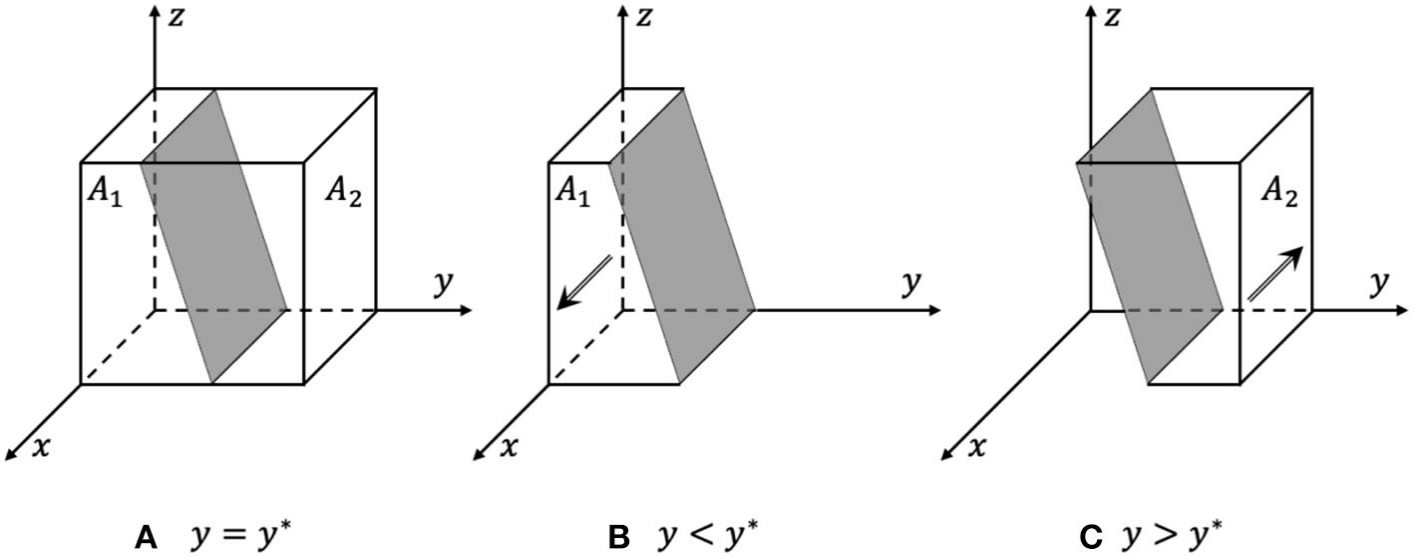
Phase diagram of the government's strategy evolution. **(A)** y = y*, all strategies are in a stable state. **(B)** y < y*, x = 1 are the evolutionarily stable strategy. **(C)** y > y*, x = 0 are the evolutionarily stable strategy.

Proof. According to Formula (3) and (10), since *G*(*y*) is an increasing function:

1. When *y* = y^*^, *G*(*y*) = 0, $\frac{dF\left(x\right)}{dx}\equiv 0$, and *x* are in an evolutionarily stable state, as shown in Figure 2A.
2. When *y* < y^*^, $G\left(y\right)\;<\;0,\;\frac{dF\left(x\right)}{dx}{|}_{x=1}\;<\;0$, and *x* = 1 are the evolutionarily stable strategy, as shown in Figure 2B; otherwise, when *y* > y^*^, *x* = 0 is the evolutionarily stable strategy, as shown in Figure 2C.

In the process of evolution, the probability that the government chooses strict supervision decreases with the increase in the probability that e-commerce platforms choose active responses and the probability that rights holders choose active rights protection. The phase diagram of the government's strategy evolution is shown in Figure 2. The volume of the probability that the government chooses strict supervision is *A*_1_, and the volume of loose supervision probability is *A*_2_.

From Formula (11) and Figure 2, it can be inferred that the probability that the government chooses strict supervision is positively related to the fines for the e-commerce platforms and platform operators, the superior authority's penalties, and the social benefits of the strict supervision. The probability is negatively related to the cost of strict supervision, the cost of rights protection for rights holders, the rewards for e-commerce platforms, and the social benefits of loose supervision.

#### Analysis of Strategy Stability of E-Commerce Platforms

Proposition 2.

1. When *x* = x^*^, all strategies are in a stable state.
2. When *x* ≠ x^*^, *F*(*y*) = 0, *y* = 0 and *y* = 1 are the stable points of *y*.

**Figure 3:**
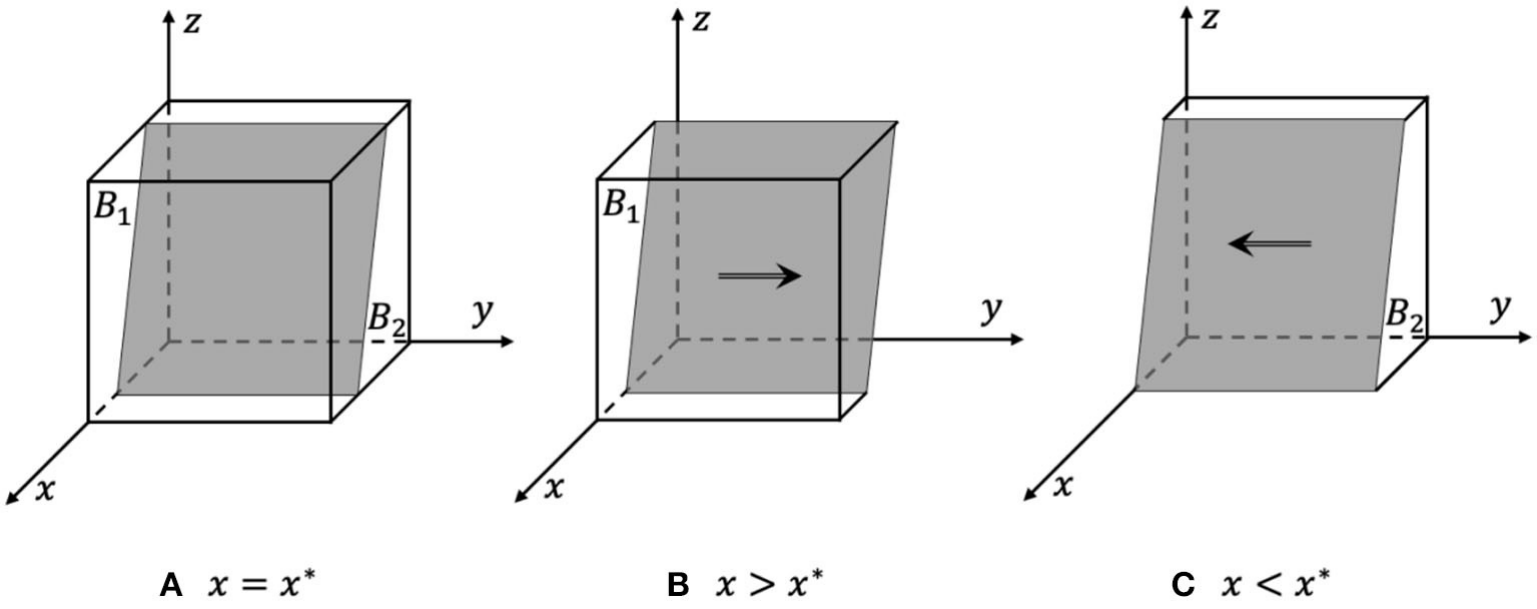
Phase diagram of e-commerce platforms' strategy evolution. **(A)** x = x*, all strategies are in a stable state. **(B)** x > x*, y = 1 are the evolutionarily stable strategy. **(C)** x < x*, y = 0 are the evolutionarily stable strategy.

Proof. According to Formula (6) and (10), since *H*(*x*) is a decreasing function:

1. When *x* = x^*^, *H*(*x*) = 0, $\frac{dF\left(y\right)}{dy}\;\equiv \;0$, and *y* are in an evolutionarily stable state, as shown in Figure 3A.
2. When *x* < x^*^, *H*(*x*) < 0, $\;\frac{dF\left(y\right)}{dy}\;{|}_{y=0}\;<\;0$, and *y* = 0 are the evolutionarily stable strategy, as shown in Figure 3C; and otherwise, when *x* > x^*^, *y* = 1 is the evolutionarily stable strategy, as shown in Figure 3B.

In the process of evolution, the probability that e-commerce platforms choose to actively respond increases with the increase in the probability that the government chooses strict supervision and the probability that the rights holders choose active rights protection. The phase diagram of e-commerce platform strategy evolution is shown in Figure 3. The volume of the probability that e-commerce platforms choose to actively respond is *B*_1_, and the volume of the passive response probability is *B*_2_.

From Formula (11) and Figure 3, it can be inferred that the probability that e-commerce platforms choose to actively respond is positively related to the cost of passively responding to infringement issues, the cost of docking between e-commerce platforms and rights holders, the rewards and fines for e-commerce platforms, and the social benefits of active responses. The probability is negatively related to the cost of active response and the social benefits of passive response.

#### Analysis of Strategy Stability of Rights Holders

Proposition 3.

1. When *y* = y^**^, all strategies are in a stable state.
2. When *y* ≠ y^**^, *F*(*z*) = 0, *z* = 0 and *z* = 1 are the stable points of *z*.

**Figure 4:**
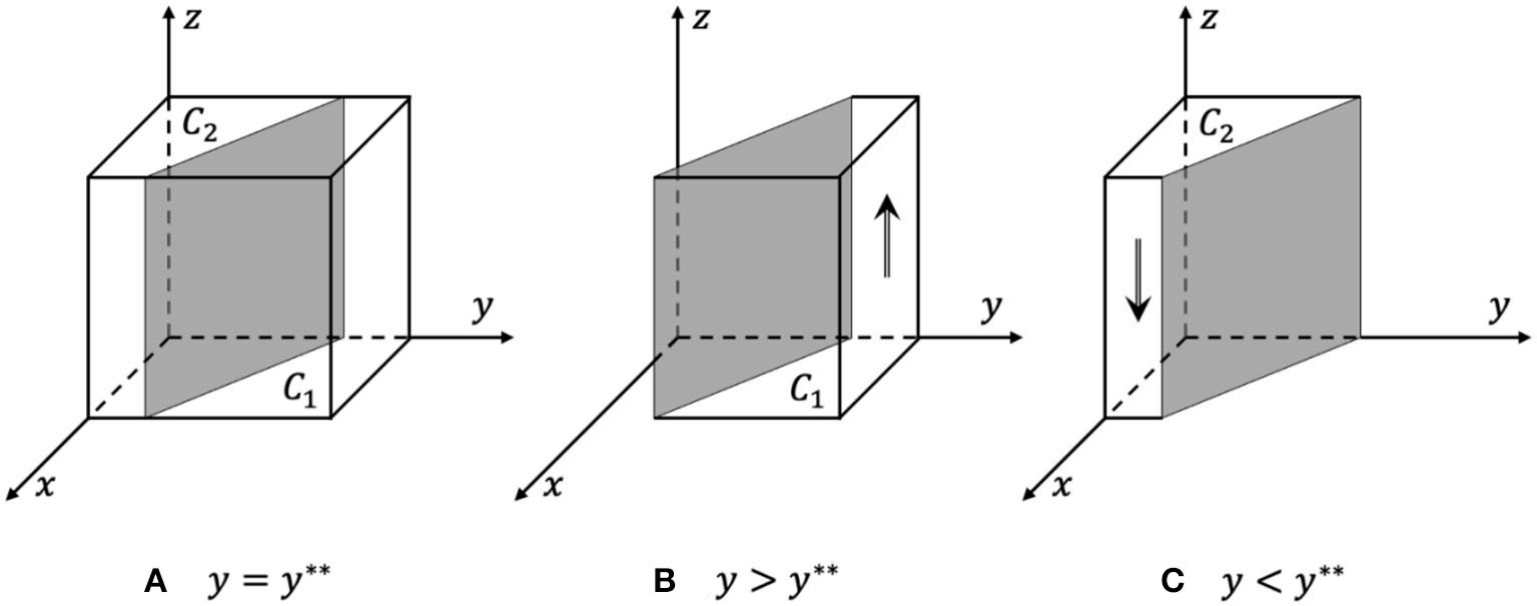
Phase diagram of rights holders' strategy evolution. **(A)** y = y**, all strategies are in a stable state. **(B)** y > y**, z = 1 are the evolutionarily stable strategy. **(C)** y < y**, z = 0 are the evolutionarily stable strategy.

Proof. According to Formula (9) and (10), since *J*(*y*) is a decreasing function:

1. When *y* = y^**^, *J*(*y*) = 0, $\frac{dF\left(z\right)}{dz}\;\equiv \;0$, and *z* are in an evolutionarily stable state, as shown in Figure 4A.
2. When *y* < y^**^, *J*(*y*) < 0, $\;\frac{dF\left(z\right)}{dz}{|}_{z=0}\;<\;0$, and *z* = 0 are the evolutionarily stable strategy, as shown in Figure 4C; otherwise, when *y* > y^**^, *z* = 1 is the evolutionarily stable strategy, as shown in Figure 4B.

In the process of evolution, the probability that rights holders choose active rights protection increases with the increase in the probability that the government chooses strict supervision and the probability that e-commerce platforms choose to actively respond. The phase diagram of rights holders' strategy evolution is shown in Figure 4. The volume of the probability that rights holders choose active rights protection is *C*_1_, and the volume of the passive rights protection probability is *C*_2_.

From Formula (11) and Figure 4, it can be inferred that the probability that rights holders choose active rights protection is positively related to the cost of passive rights protection, the loss when rights holders passively defend their rights, and the response cost of the strict supervision by the government. The probability is negatively related to the cost of active rights protection, the loss when rights holders take the initiative to defend their rights, and the cost of docking between e-commerce platforms and rights holders.

### Stability Analysis of Equilibrium Points

According to the local stability analysis, let *F*(*x*) = 0, *F*(*y*) = 0, and *F*(*z*) = 0, respectively. We can obtain the equilibrium points of the replicator dynamic equations, which are *U*_1_(0, 0, 0), *U*_2_(1, 0, 0), *U*_3_(0, 1, 0), *U*_4_(0, 0, 1), *U*_5_(1, 1, 0), *U*_6_(1, 0, 1), *U*_7_(0, 1, 1), and *U*_8_(1, 1, 1). $U_{9}\left(x^{*},\;y^{*},\;z^{*}\right)$ is the solution to

(12) $$\begin{array}{l} \{\begin{array}{c} y\left(M_{p}\;+\;P_{p}\;+\;P_{g}\right)\;+\;zC_{gh}\;+\;(C_{g}\;-\;P_{p}\;-\;P_{s}\;-\;P_{g}\\ -\;S_{g}\;+\;L_{g})=0\\ -x\left(M_{p}\;+\;P_{p}\right)\;-\;zC_{ph}\;+\;(C_{pa}\;-\;C_{pp}\\ -\;S_{p}\;+\;L_{p})=0\\ -xC_{gh}\;-\;yC_{ph}\;+\;(C_{ha}\;-\;C_{hp}\;+\;C_{ph}\\ +\;G_{ha}\;-\;G_{hp})=0 \end{array} \end{array}$$

Based on the research findings of Friedman, the stability of the equilibrium points is derived from analyzing the Jacobian matrix. In addition, the Jacobian matrix can be obtained as follows:

(13) $$\begin{array}{l} J=\left[\begin{array}{ccc} J_{1}\; & \;J_{2}\; & \;J_{3}\\ J_{4}\; & \;J_{5}\; & \;J_{6}\\ J_{7}\; & \;J_{8}\; & \;J_{9} \end{array}\right] \end{array}$$

where

$$\begin{array}{l} J_{1}=\frac{\partial F\left(x\right)}{\partial x}=\left(2x\;-\;1\right)[y\left(M_{p}\;+\;P_{p}\;+\;P_{g}\right)\\ \;+zC_{gh}\;+\;\left(C_{g}\;-\;P_{p}\;-\;P_{s}\;-\;P_{g}\;-\;S_{g}\;+\;L_{g}\right)],\\ J_{2}=\frac{\partial F\left(x\right)}{\partial y}=x\left(x\;-\;1\right)\left(M_{p}\;+\;P_{p}\;+\;P_{g}\right),\\ J_{3}=\frac{\partial F\left(x\right)}{\partial z}=x\left(x\;-\;1\right)C_{gh},\\ J_{4}=\frac{\partial F\left(y\right)}{\partial x}=-y\left(y\;-\;1\right)\left(M_{p}\;+\;P_{p}\right), \end{array}$$

(14) $$\begin{array}{l} J_{5}=\frac{\partial F\left(y\right)}{\partial y}=\left(2y\;-\;1\right)[-x\left(M_{p}\;+\;P_{p}\right)\;-\;zC_{ph}\\ \;+\;\left(C_{pa}\;-\;C_{pp}\;-\;S_{p}\;+\;L_{p}\right)]],\\ J_{6}=\frac{\partial F\left(y\right)}{\partial z}=-y\left(y\;-\;1\right)C_{ph},\\ J_{7}=\frac{\partial F\left(z\right)}{\partial x}=-z\left(z\;-\;1\right)C_{gh},\\ J_{8}=\frac{\partial F\left(z\right)}{\partial y}=-z\left(z\;-\;1\right)C_{ph},\\ J_{9}=\frac{\partial F\left(z\right)}{\partial z}=\left(2z\;-\;1\right)[-xC_{gh}\;-\;yC_{ph}\;+\;(C_{ha}\;-\;C_{hp}\;\\ \;+C_{ph}\;+\;G_{ha}\;-\;G_{hp})] \end{array}$$

Based on Lyapunov stability analysis, considering that the equilibrium solution of the tripartite evolutionary game follows a strict Nash equilibrium, we do not consider *U*_9_. When the eigenvalues of the matrix *J* are all positive, the equilibrium point is an unstable point; when the eigenvalues of matrix *J* have positive values, the equilibrium point is a saddle point; when the eigenvalues are all negative, the equilibrium point is an evolutionarily stable strategy. The main eigenvalues of the Jacobian matrix with different equilibrium points are shown in Table 3.

**Table 3:** The eigenvalues of the Jacobian matrix.

**x, y, z**	**λ_1_, λ_2_, λ_3_**	**Case**
0, 0, 0	− *C*_*g*_ + *P*_*p*_ + *P*_*g*_ + *S*_*g*_ − *L*_*g*_, − *C*_*pa*_ + *C*_*pp*_ + *S*_*p*_ − *L*_*p*_, − *C*_*ha*_ + *C*_*hp*_ − *C*_*ph*_ − *G*_*ha*_ + *G*_*hp*_	①
1, 0, 0	*C*_*g*_ − *P*_*p*_ − *P*_*s*_ − *P*_*g*_ − *S*_*g*_ + *L*_*g*_, − *C*_*pa*_ + *C*_*pp*_ + *M*_*p*_ + *P*_*p*_ + *S*_*p*_ − *L*_*p*_, − *C*_*ha*_ + *C*_*hp*_ + *C*_*gh*_ − *C*_*ph*_ − *G*_*ha*_ + *G*_*hp*_	②
0, 1, 0	− *C*_*g*_ − *M*_*p*_ + *P*_*s*_ + *S*_*g*_ − *L*_*g*_, *C*_*pa*_ − *C*_*pp*_ − *S*_*p*_ + *L*_*p*_, − *C*_*ha*_ + *C*_*hp*_ − *G*_*ha*_ + *G*_*hp*_	③
0, 0, 1	− *C*_*g*_ − *C*_*gh*_ + *P*_*p*_ + *P*_*g*_ + *S*_*g*_ − *L*_*g*_, − *C*_*pa*_ + *C*_*pp*_ + *C*_*ph*_ + *S*_*p*_ − *L*_*p*_, *C*_*ha*_ − *C*_*hp*_ + *C*_*ph*_ + *G*_*ha*_ − *G*_*hp*_	④
1, 1, 0	*C*_*g*_ + *M*_*p*_ − *P*_*s*_ − *S*_*g*_ + *L*_*g*_, *C*_*pa*_ − *C*_*pp*_ − *M*_*p*_ − *P*_*p*_ − *S*_*p*_ + *L*_*p*_, − *C*_*ha*_ + *C*_*hp*_ + *C*_*gh*_ − *G*_*ha*_ + *G*_*hp*_	⑤
1, 0, 1	*C*_*g*_ + *C*_*gh*_ − *P*_*p*_ − *P*_*s*_ − *P*_*g*_ − *S*_*g*_ + *L*_*g*_, − *C*_*pa*_ + *C*_*pp*_ + *C*_*ph*_ + *M*_*p*_ + *P*_*p*_ + *S*_*p*_ − *L*_*p*_, *C*_*ha*_ − *C*_*hp*_ − *C*_*gh*_ + *C*_*ph*_ + *G*_*ha*_ − *G*_*hp*_	⑥
0, 1, 1	− *C*_*g*_ − *C*_*gh*_ − *M*_*p*_ + *P*_*s*_ + *S*_*g*_ − *L*_*g*_, *C*_*pa*_ − *C*_*pp*_ − *C*_*ph*_ − *M*_*p*_ − *S*_*p*_ + *L*_*p*_, *C*_*ha*_ − *C*_*hp*_ + *G*_*ha*_ − *G*_*hp*_	⑦
1, 1, 1	*C*_*g*_ + *C*_*gh*_ + *M*_*p*_ − *P*_*s*_ − *S*_*g*_ + *L*_*g*_, *C*_*pa*_ − *C*_*pp*_ − *C*_*ph*_ − *M*_*p*_ − *P*_*p*_ − *S*_*p*_ + *L*_*p*_, *C*_*ha*_ − *C*_*hp*_ − *C*_*gh*_ + *G*_*ha*_ − *G*_*hp*_	⑧

## Simulation Analysis

To verify the validity of the model-related analysis and propositions, the model is assigned numerical values based on the actual situation, and MATLAB R2017b is used for numerical simulation.

**Array 1:** We set *C*_*g*_ = 100, *P*_*s*_ = 10, *P*_*g*_ = 10, *S*_*g*_ = 20, *L*_*g*_ = 10, *C*_*pa*_ = 150, *C*_*pp*_ = 50, *M*_*p*_ = 10, *P*_*p*_ = 10, *S*_*p*_ = 20, *L*_*p*_ = 10, *C*_*ha*_ = 100, *C*_*hp*_ = 50, *C*_*ph*_ = 50, *C*_*gh*_ = 20, *G*_*ha*_ = 10, and *G*_*hp*_ = 50. At this time, the system satisfies Case 1 in Table 3, and the strategy set is (loose supervision, passive response, passive rights protection). This is the nonideal state of the system.

### Effect of P_p_ on Strategy Selection

**Figure 5:**
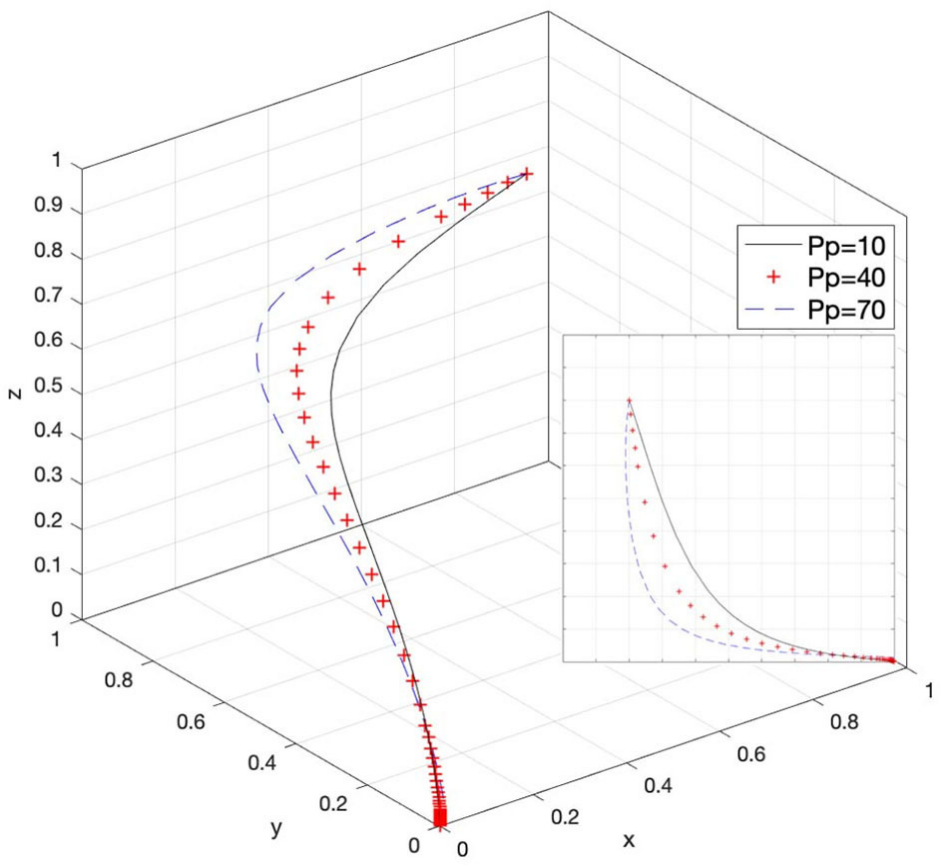
Effect of the fines for e-commerce platforms' passive response.

Let *P*_*p*_ = 10, *P*_*p*_ = 40, and *P*_*p*_ = 70; then, the system evolution simulation result is shown in Figure 5.

Figure 5 shows that in the process of the system evolving and reaching a stable state, the fines for e-commerce platforms' passive response inhibit the evolution speed of the government's choice of loose supervision strategy. In other words, as the value of *P*_*p*_ increases, the government's willingness to choose strict supervision increases. Therefore, reasonably regulating the penalties for e-commerce platforms' passive response to infringement issues under the government's strict supervision can increase the enthusiasm of government supervisory agencies for strict supervision and provide sufficient incentives to them. At the same time, it will also reduce the willingness of e-commerce platforms to passively respond to infringement issues and give them a certain amount of deterrence.

### Effect of S_g_ and L_g_ on Strategy Selection

**Figure 6:**
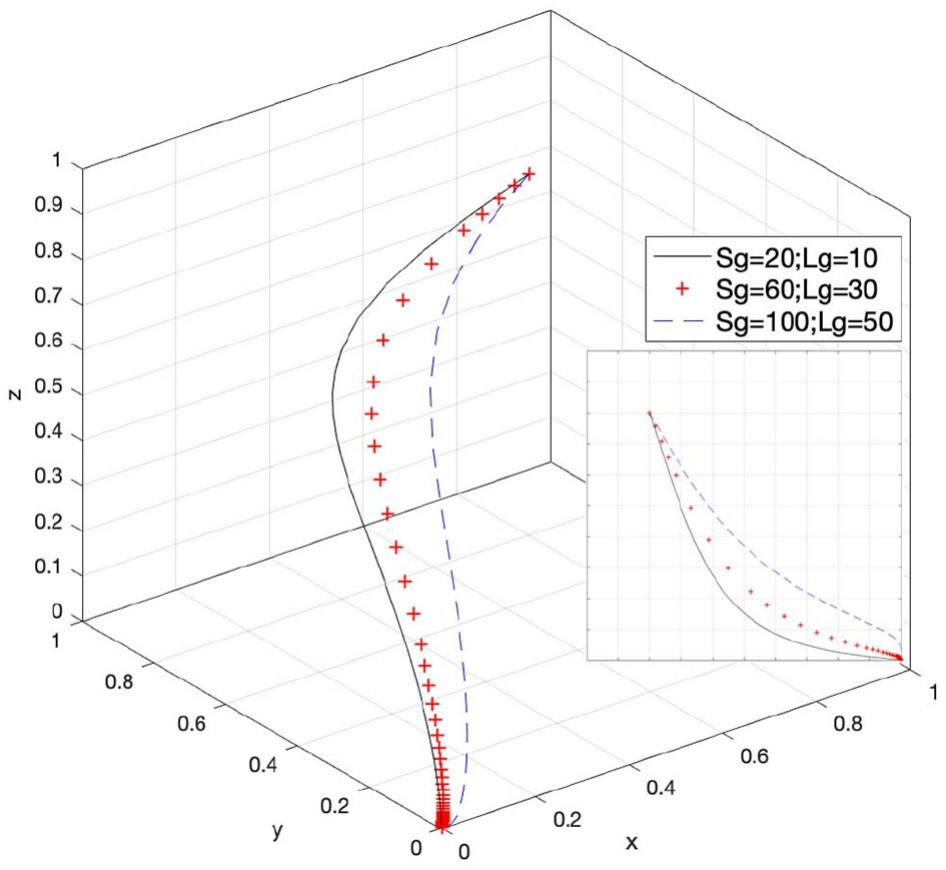
Effect of the social benefits of the government's supervision.

Let *S*_*g*_ = 20 and *L*_*g*_ = 10, *S*_*g*_ = 60 and *L*_*g*_ = 30, and *S*_*g*_ = 100 and *L*_*g*_ = 50; then, the system evolution simulation result is shown in Figure 6.

Figure 6 shows that in the process of the system evolving and reaching a stable state, the social benefits of the government's supervision promote the evolution speed of the government's choice of loose supervision strategy. However, as the value of (*S*_*g*_ − *L*_*g*_) increases, the state of loose supervision by government supervisory agencies is more difficult to stabilize. Therefore, boosting the society's emphasis on intellectual property rights, promoting the overall supervision of the government supervisory agencies, increasing the social benefit value obtained by the government's strict supervision, and keeping the gap between it and the social benefit value obtained during loose supervision at a certain high level will help increase the willingness of the government to strictly supervise.

### Effect of S_p_ and L_p_ on Strategy Selection

**Figure 7:**
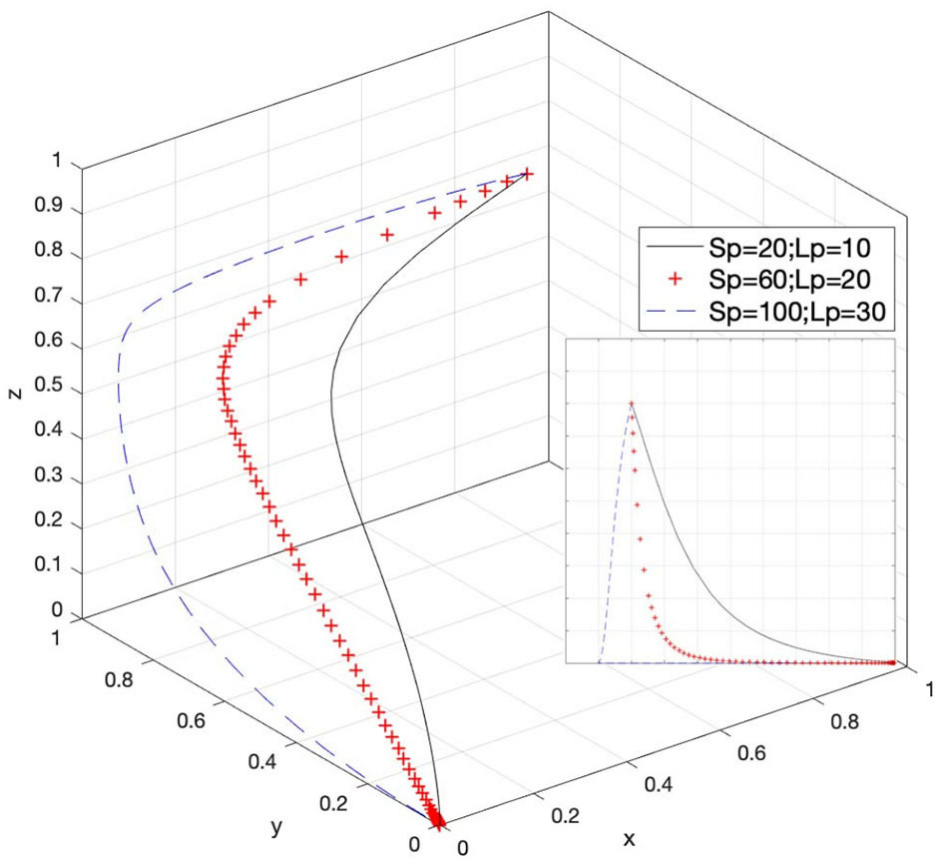
Effect of the social benefits of e-commerce platforms' response attitudes.

Let *S*_*p*_ = 20 and *L*_*p*_ = 10, *S*_*p*_ = 60 and *L*_*p*_ = 20, and *S*_*p*_ = 100 and *L*_*p*_ = 30; then, the system evolution simulation result is shown in Figure 7.

Figure 7 shows that in the process of the system evolving and reaching a stable state, the social benefits of e-commerce platforms' response attitudes inhibit the evolution speed of e-commerce platforms' choice of passively response strategy. As the value of (*S*_*p*_ − *L*_*p*_) increases, the state of passive response is more difficult to stabilize. In other words, the probability of e-commerce platforms actively responding to infringement issues will increase. Therefore, a reasonable increase in the value of social benefits obtained by e-commerce platforms in responding to infringement issues can boost platforms' confidence, and increasing the social benefits value obtained by e-commerce platforms in actively responding to infringement issues can promote e-commerce platforms to increase their active response to infringement issues compared to passive response.

### Effect of C_ph_ on Strategy Selection

**Figure 8:**
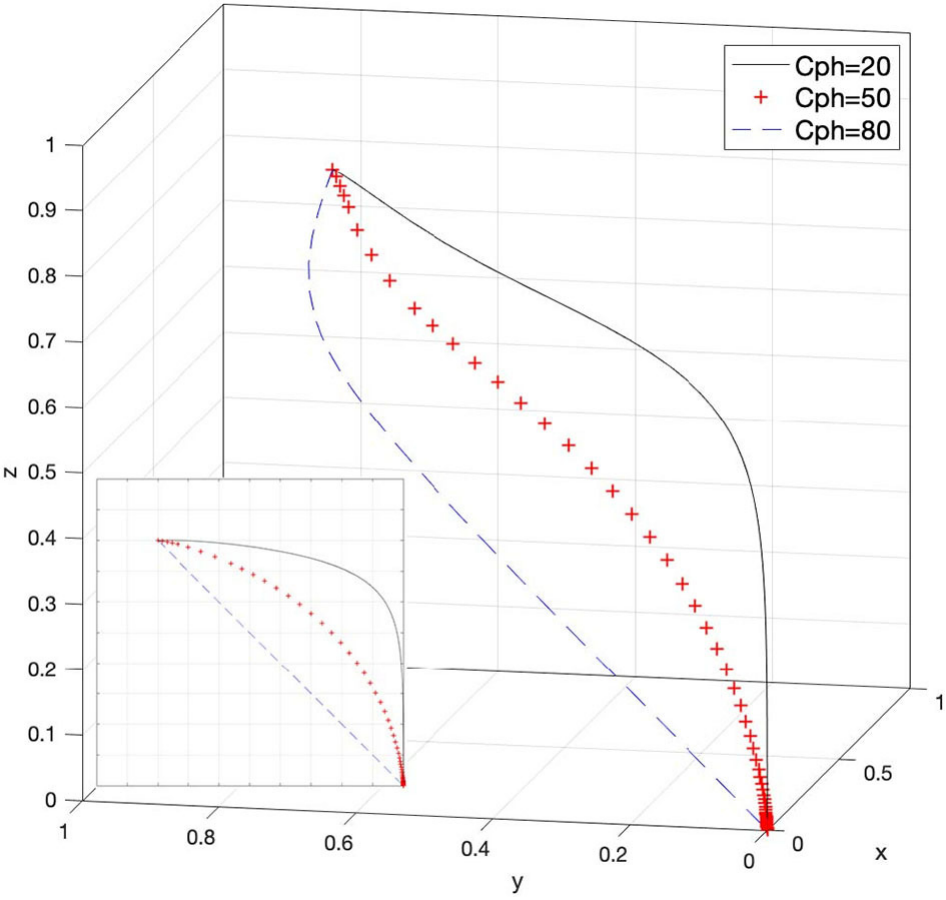
Effect of the cost of docking between rights holders and e-commerce platforms.

Let *C*_*ph*_ = 20, *C*_*ph*_ = 50, and *C*_*ph*_ = 80; then, the system evolution simulation result is shown in Figure 8.

Figure 8 shows that in the process of the system evolving and reaching a stable state, the increase in the cost of docking between rights holders and e-commerce platforms promotes the evolution speed of rights holders' choice of passive rights protection strategy. In other words, as the value of *C*_*ph*_ increases, rights holders' willingness to actively protect rights will be greatly reduced. Therefore, rationally regulating the cost of docking between the two or urging the e-commerce platforms to actively respond to infringement issues to eliminate costs, can effectively increase the willingness of rights holders to choose to actively protect their rights and participate in the social co-governance system of intellectual property protection. Corresponding viewpoints can also be drawn from Case 2, Case 4 and Case 6 in Table 3.

### Effect of G_ha_ and G_hp_ on Strategy Selection

**Figure 9:**
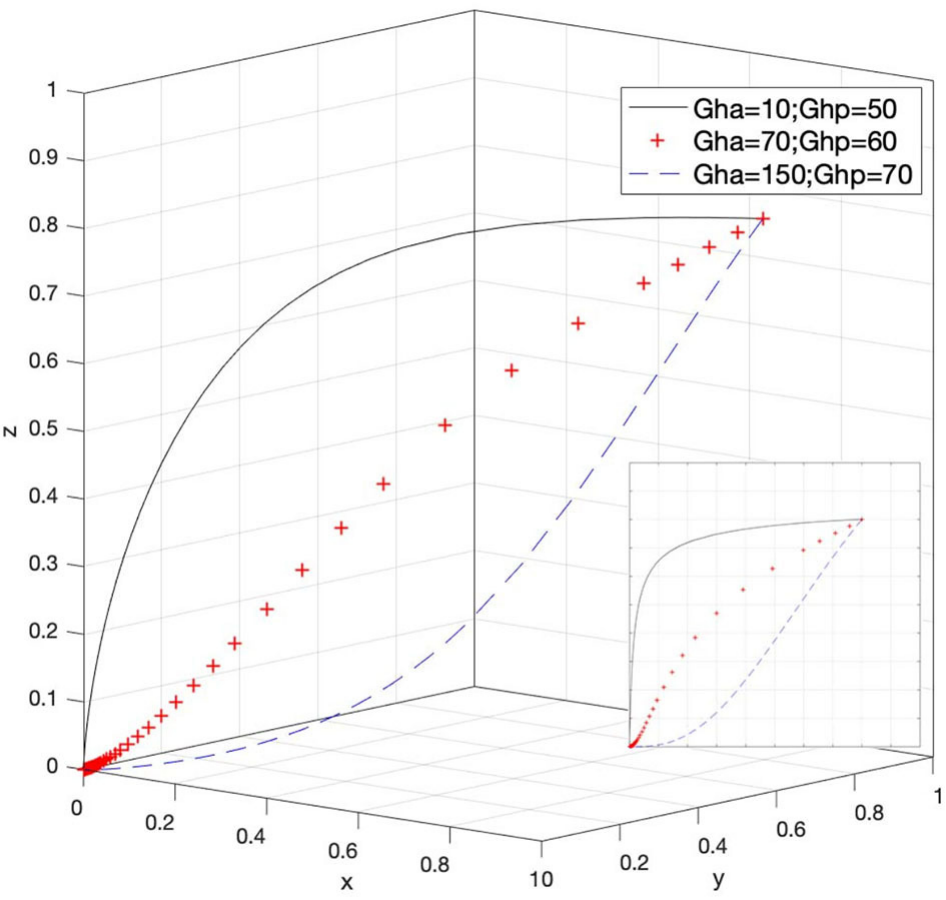
Effect of the loss of rights holders' active and passive rights protection.

Let *G*_*ha*_ = 10 and *G*_*hp*_ = 50, *G*_*ha*_ = 70 and *G*_*hp*_ = 60, and *G*_*ha*_ = 150 and *G*_*hp*_ = 70; then, the system evolution simulation result is shown in Figure 9.

Figure 9 shows that in the process of the system evolving and reaching a stable state; the loss difference between the active and passive rights protection of rights holders affects the evolution speed. When *G*_*ha*_ − *G*_*hp*_ < 0, it is relatively difficult for rights holders to choose passive rights protection to form a stable state. At this time, rights holders will be more likely to choose active rights protection; when *G*_*ha*_ − *G*_*hp*_ > 0, rights holders are more willing to choose to wait passively rather than take the initiative to attack. Therefore, reducing, retrieving, or avoiding the loss when rights holders choose to actively protect their rights and enhancing the sense of gain when rights holders actively protect their rights will help increase the willingness of rights holders to actively protect their legitimate rights and interests.

**Array 2:** We set *C*_*g*_ = 100, *P*_*s*_ = 10, *P*_*g*_ = 40, *S*_*g*_ = 40, *L*_*g*_ = 10, *C*_*pa*_ = 150, *C*_*pp*_ = 50, *M*_*p*_ = 10, *P*_*p*_ = 40, *S*_*p*_ = 10, *L*_*p*_ = 10, *C*_*ha*_ = 100, *C*_*hp*_ = 50, *C*_*ph*_ = 10, *C*_*gh*_ = 20, *G*_*ha*_ = 50, and *G*_*hp*_ = 50. At this time, the system satisfies Case 2 in Table 3, and the strategy set is {strict supervision, passive response, passive rights protection}. This is the nonideal state of the system.

### Effect of C_g_ on Strategy Selection

**Figure 10:**
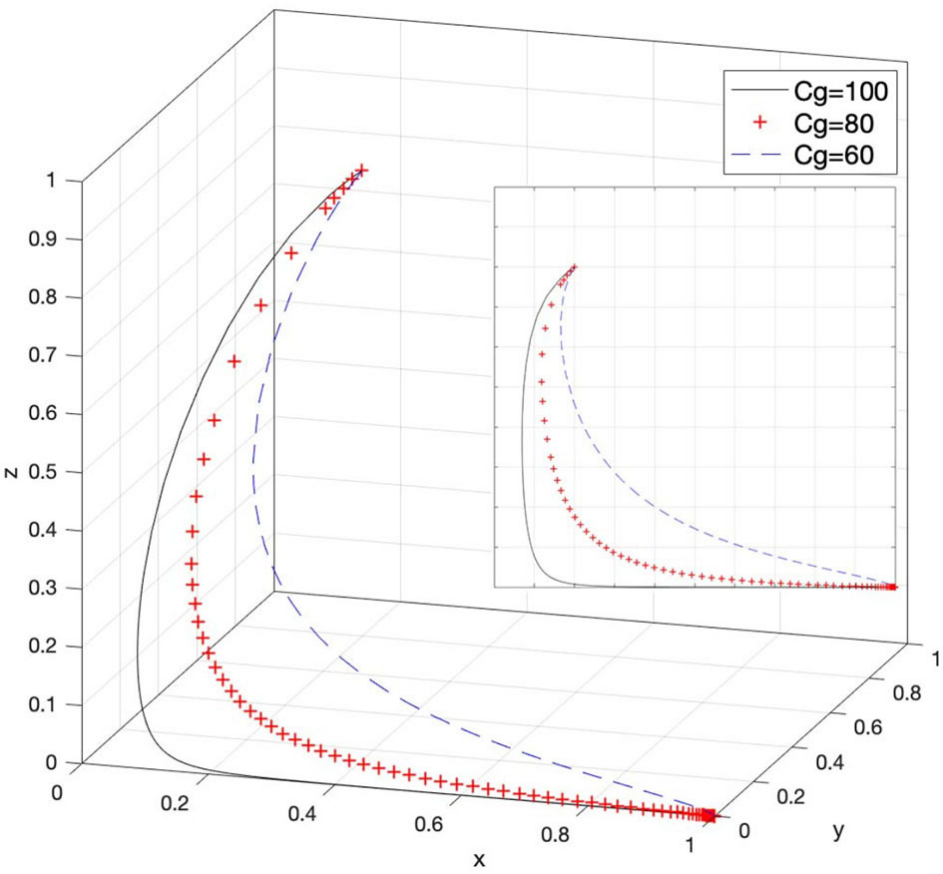
Effect of the cost of strict supervision by the government.

Let *C*_*g*_ = 100, *C*_*g*_ = 80, and *C*_*g*_ = 60; then, the system evolution simulation result is shown in Figure 10.

Figure 10 shows that in the process of the system evolving and reaching a stable state, the cost of strict supervision by the government inhibits the evolution speed of the government's choice of strict supervision strategy. In other words, as the value of *C*_*g*_ decreases, the strict supervision strategy of the government can reach a stable state faster. Therefore, multiangle and comprehensive control of the cost of the government's strict supervision activities can greatly enhance the supervision enthusiasm of government supervisory agencies and strengthen their supervision. In addition, it can also provide a deterrent to possible intellectual property infringements in e-commerce platforms.

### Effect of M_p_ and P_p_ on Strategy Selection

**Figure 11:**
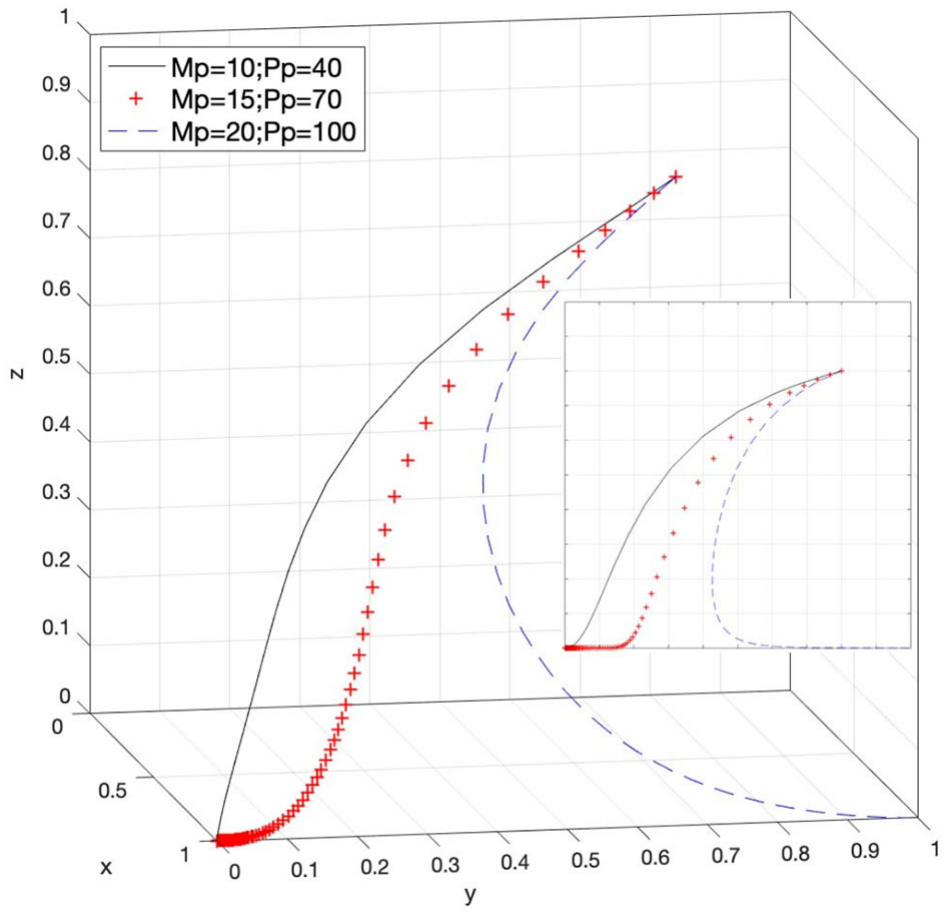
Effect of rewards and punishments for different positive attitudes toward e-commerce platforms under strict government supervision.

Let *M*_*p*_ = 10 and *P*_*p*_ = 40, *M*_*p*_ = 15 and *P*_*p*_ = 70, and *M*_*p*_ = 20 and *P*_*p*_ = 100; then, the system evolution simulation result is shown in Figure 11.

Figure 11 shows that in the process of the system evolving and reaching a stable state, under the strict supervision of the government, the rewards and punishments for e-commerce platforms to actively or passively respond to infringement issues inhibit the evolution speed. As the values of *M*_*p*_ and *P*_*p*_ increase to a certain amount, the choice of the government changes from a stable state of loose supervision to strict supervision (that is, Case 5 in Table 3 is satisfied). Therefore, reasonable control of the reward amount for e-commerce platforms to actively respond to infringement issues and increase in the penalty amount for passive response issues help boost the government's enthusiasm for strict supervision during strict government supervision. This has also improved the degree of active response of e-commerce platforms to infringement issues and will help deter infringements within the platform. Corresponding viewpoints can also be drawn from Case 6 and Case 8 in Table 3.

### Effect of C_gh_ on Strategy Selection

**Figure 12:**
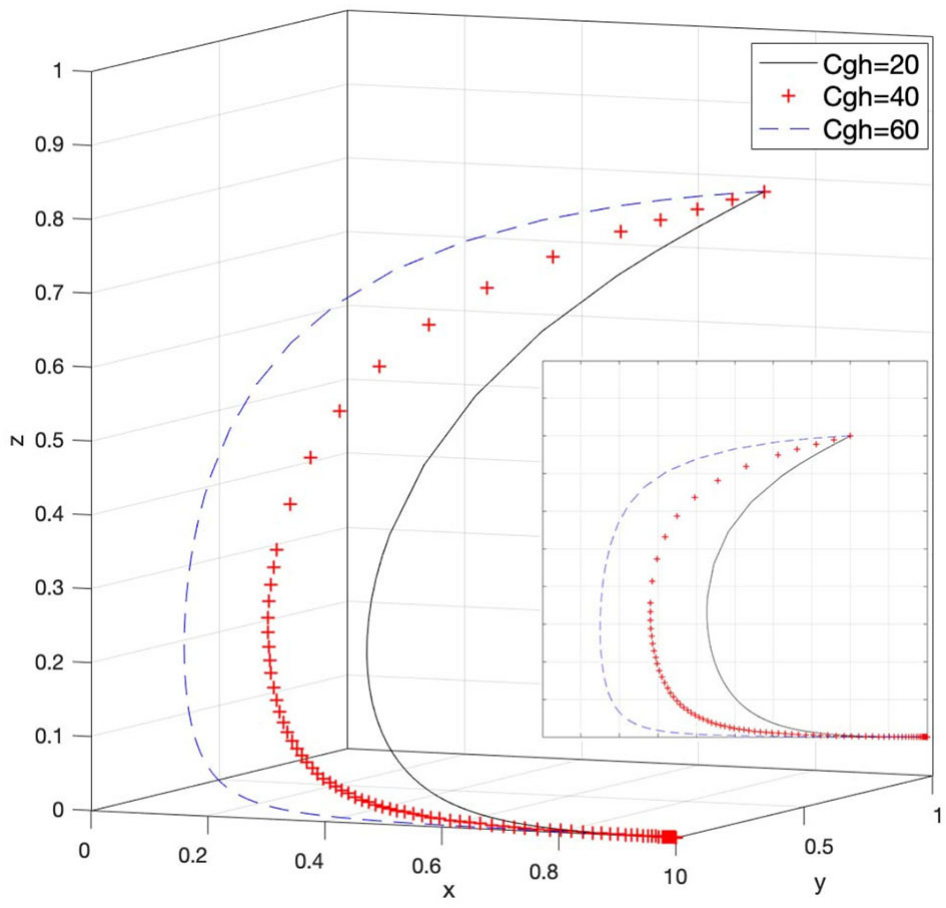
Effect of the response cost on rights holders when rights holders actively protect their rights, and the government chooses strict supervision.

Let *C*_*gh*_ = 20, *C*_*gh*_ = 40, and *C*_*gh*_ = 60; then, the system evolution simulation result is shown in Figure 12.

Figure 12 shows that in the process of the system evolving and reaching a stable state, the response cost of the government when rights holders actively protect their rights, and the government chooses strict supervision inhibits the evolution speed of rights holders to passively protect their rights. In other words, as the value of *C*_*gh*_ continues to increase, rights holders are closer to choosing to actively protect their rights. At this time, it is not difficult to find that the government is more inclined to choose a loose supervision strategy. Therefore, the strict supervision by government supervisory agencies under the attitude of rights holders to actively defend their rights also needs to control response costs. We should reasonably adjust the response cost to the government when rights holders actively protect their rights. Only in this way, while promoting strict supervision by the government regulatory agencies, can rights holders be urged to take the initiative to protect their legitimate rights and interests. The change in the value of *C*_*gh*_ in Case 5 in Table 3 can also reflect the above content.

**Array 3:** We set *C*_*g*_ = 100, *P*_*s*_ = 10, *P*_*g*_ = 40, *S*_*g*_ = 40, *L*_*g*_ = 10, *C*_*pa*_ = 70, *C*_*pp*_ = 50, *M*_*p*_ = 10, *P*_*p*_ = 40, *S*_*p*_ = 50, *L*_*p*_ = 10, *C*_*ha*_ = 100, *C*_*hp*_ = 50, *C*_*ph*_ = 10, *C*_*gh*_ = 20, *G*_*ha*_ = 50, and *G*_*hp*_ = 50. At this time, the system satisfies Case 3 in Table 3, and the strategy set is (loose supervision, active response, passive rights protection). This is the non-ideal state of the system.

### Effect of M_p_ and P_s_ on Strategy Selection

**Figure 13:**
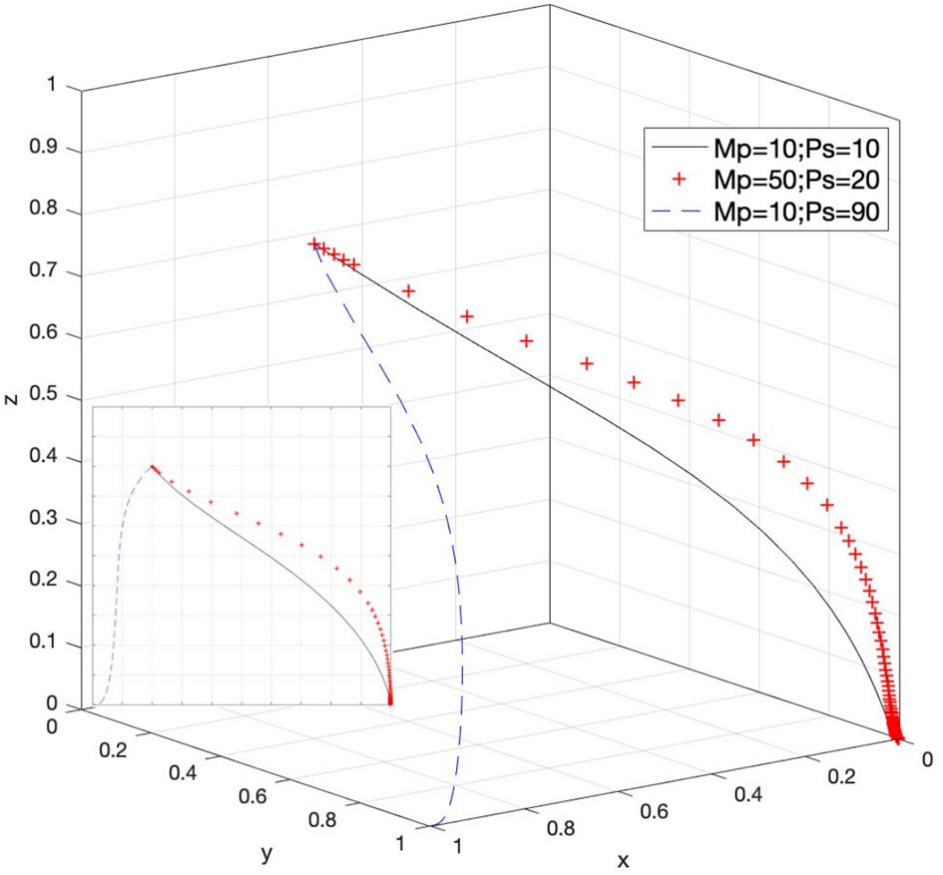
Effects of the rewards for e-commerce platforms' positive attitude and the fines for infringements within platforms when the government chooses strict supervision.

Let *M*_*p*_ = 10 and *P*_*s*_ = 10, *M*_*p*_ = 50 and *P*_*s*_ = 20, and *M*_*p*_ = 10 and *P*_*s*_ = 90; then, the system evolution simulation result is shown in Figure 13.

Figure 13 shows that in the process of the system evolving and reaching a stable state, under the strict supervision attitude of the government supervisory agencies, the rewards to e-commerce platforms for actively responding to infringement issues and the fines for infringements of platform operators affect the attitude selection of the government. In this situation, when *M*_*p*_ and *P*_*s*_ increase, they have a certain inhibitory effect on the stable attitude of the government's loose supervision. When the added value of *P*_*s*_ compared to *M*_*p*_ reaches a certain amount, the strategic choice of the government supervisory agencies will change from loose supervision to strict supervision; that is, the state of Case 5 in Table 3 is satisfied. Therefore, rationally regulating the rewards of the government for e-commerce platforms and appropriately strengthening the penalties for infringements that occur within the platform will help increase the willingness of the government to choose strict supervision. This imposes a certain degree of deterrence on the possible infringements of platform operators. In addition, Case 7 and Case 8 in Table 3 can also reflect this set of changes.

**Array 4:** We set *C*_*g*_ = 100, *P*_*s*_ = 10, *P*_*g*_ = 50, *S*_*g*_ = 40, *L*_*g*_ = 10, *C*_*pa*_ = 150, *C*_*pp*_ = 50, *M*_*p*_ = 10, *P*_*p*_ = 10, *S*_*p*_ = 50, *L*_*p*_ = 60, *C*_*ha*_ = 60, *C*_*hp*_ = 50, *C*_*ph*_ = 20, *C*_*gh*_ = 20, *G*_*ha*_ = 50, and *G*_*hp*_ = 100. At this time, the system satisfies Case 4 in Table 3, and the strategy set is {loose supervision, passive response, active rights protection}. This is the nonideal state of the system.

### Effect of P_g_ on Strategy Selection

**Figure 14:**
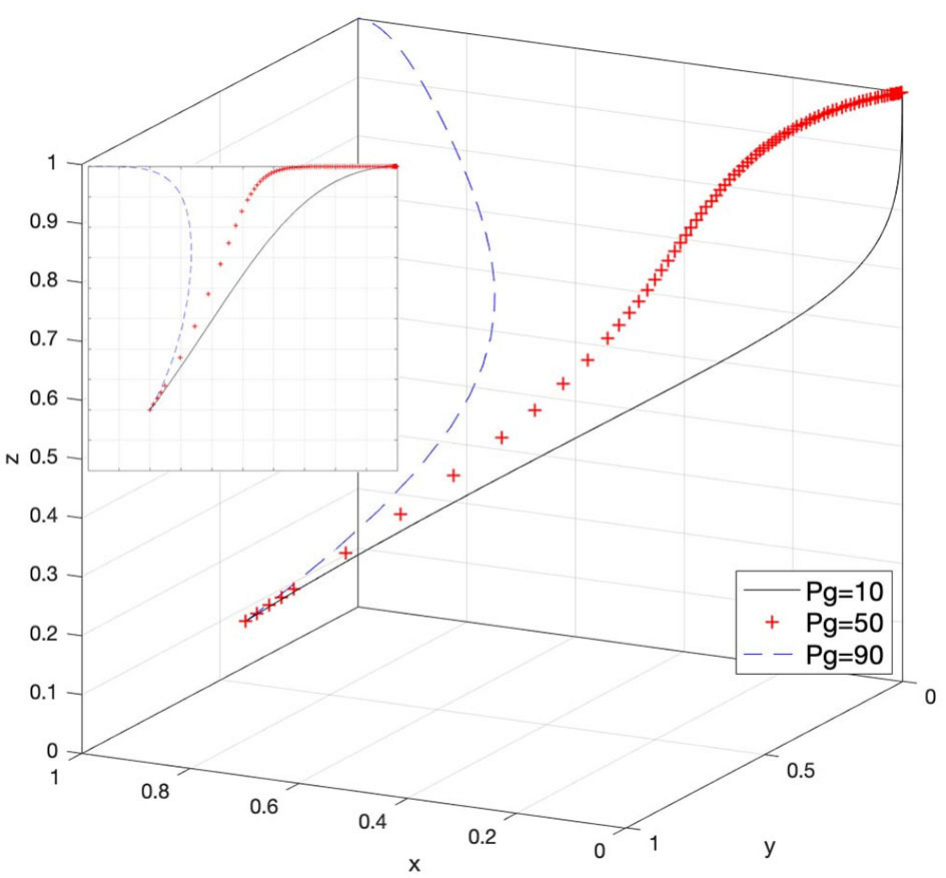
Effect of the superior authority's penalties for the government.

Let *P*_*g*_ = 10, *P*_*g*_ = 50, and *P*_*g*_ = 90; then, the system evolution simulation result is shown in Figure 14.

Figure 14 shows that in the process of the system evolving and reaching a stable state, when the e-commerce platforms passively respond to infringement issues, the government chooses loose supervision and leads to a disadvantage for intellectual property protection, the superior authority's penalties for the government promote the evolution speed of the government's choice of strict supervision strategy. As the value of *P*_*g*_ continues to increase, the loose supervision strategy of the government has difficulty achieving a stable state, and when the value of *P*_*g*_ reaches a certain amount, the system will evolve to a strict supervision strategy (that is, satisfy Case 6 in Table 3). Therefore, it is possible to enhance the supervision enthusiasm and initiative of the government by strengthening the penalty limit for the loose supervision state of the government.

**Array 5:** We set *C*_*g*_ = 100, *P*_*s*_ = 10, *P*_*g*_ = 50, *S*_*g*_ = 40, *L*_*g*_ = 10, *C*_*pa*_ = 150, *C*_*pp*_ = 50, *M*_*p*_ = 10, *P*_*p*_ = 50, *S*_*p*_ = 50, *L*_*p*_ = 60, *C*_*ha*_ = 60, *C*_*hp*_ = 50, *C*_*ph*_ = 20, *C*_*gh*_ = 20, *G*_*ha*_ = 50, and *G*_*hp*_ = 100. At this time, the system satisfies Case 6 in Table 3, and the strategy set is {strict supervision, passive response, active rights protection}. This is a nonideal state of the system.

### Effect of C_gh_ on Strategy Selection

**Figure 15:**
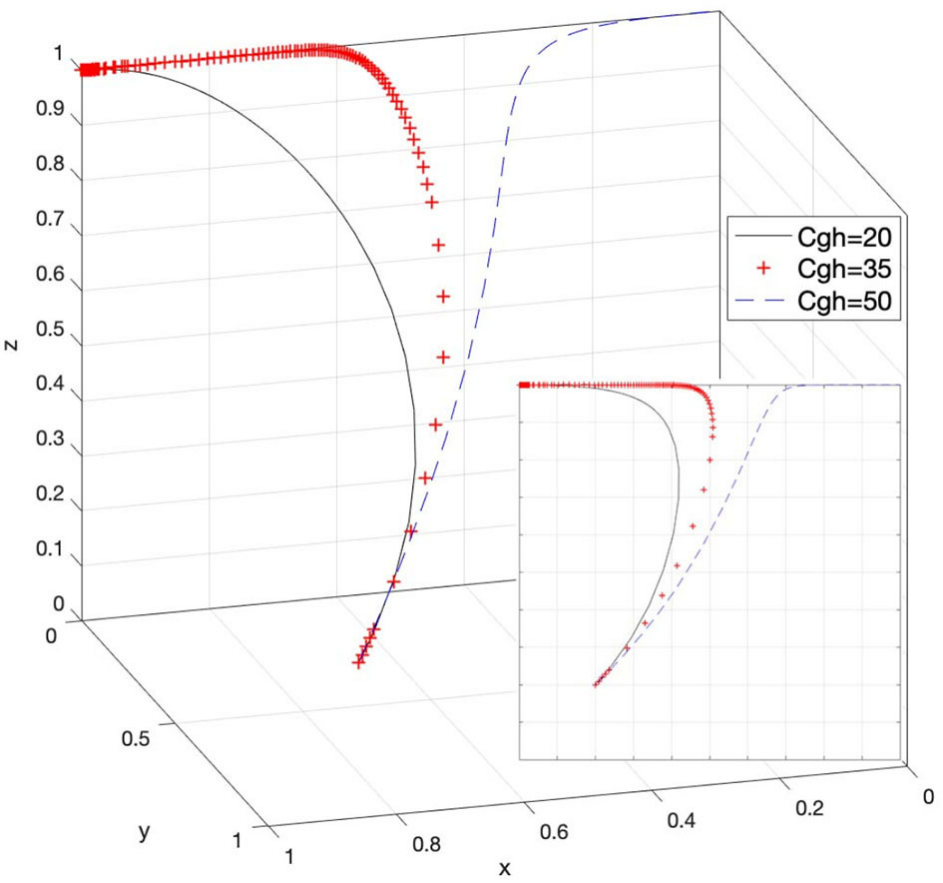
Effect of the response cost on the government when rights holders actively protect their rights, and the government chooses strict supervision.

Let *C*_*gh*_ = 20, *C*_*gh*_ = 35, and *C*_*gh*_ = 50; then, the system evolution simulation result is shown in Figure 15.

Figure 15 shows that in the process of the system evolving and reaching a stable state, the response cost when rights holders actively protect their rights, and the government chooses strict supervision promotes the evolution speed of the government's choice of strict supervision strategy. As the value of *C*_*gh*_ continues to increase, the strategy of strict supervision by the government has difficulty achieving a stable state. Therefore, it is necessary to reasonably control the cost of the government in responding to rights holders actively protecting their rights and interests and ensure that the willingness of the government to strictly supervise does not change easily. In addition, the changes in *C*_*gh*_ in Case 4 and Case 8 in Table 3 can also reflect the above content.

## Suggestions and Conclusions

### Model Analysis

Combining the above content and numerical simulation results, the evolutionary game model of the e-commerce intellectual property protection's social co-governance composed of the government, e-commerce platforms and rights holders can provide the basis for the following analysis of the three parties' strategic choices.

#### Analysis of the Government

For government supervisory agencies, effectively controlling the cost of strict supervision and stabilizing the cost at a relatively low level can enhance their preference for strict supervision. Especially when rights holders choose to actively protect their rights and interests, if the government's response costs to rights holders' rights protection actions are too high, then it will also hinder the government's choice of strict supervision strategy. Therefore, the response cost should be controlled within a reasonable limit to enhance the government's enthusiasm and stability of strict supervision.

In addition, when the government imposes a high penalty on the platform under strict supervision, it can increase the government's willingness to choose a strict supervision strategy; imposing a fine on the infringement of platform operators can allow the government to obtain certain benefits and ensure that it adopts strict supervision measures; and excessive amounts of rewards for e-commerce platforms will limit the probability of strict government supervision. At the same time, a series of punishment measures, such as the responsibility investigation and the superior authority's penalties for the government supervisory agencies due to ineffective protection of intellectual property rights, can also prompt the government to conduct strict supervision. Strengthening related penalties can effectively avoid the government's loose supervision strategy.

From the perspective of social co-governance, further optimizing the social benefit value of the government in different attitudes, the increase of the social benefit value of strict supervision or the decrease of the social benefit value of loose supervision can also prompt the government to carry out strict supervision.

#### Analysis of E-Commerce Platforms

For e-commerce platforms, under the ideal state of strict government supervision and active protection of rights and interests by rights holders, the cost of responding to infringement issues and the cost of docking with rights holders of e-commerce platforms in a positive response attitude should be controlled within a reasonable range, which can effectively enhance the enthusiasm of e-commerce platforms.

In addition, if the government sets higher incentives and penalties for e-commerce platforms, it can enhance and stabilize the willingness of e-commerce platforms to actively respond to infringement issues and reverse their tendency to passively respond to infringement issues. At the same time, the incentives and penalties should also be set reasonably in consideration of the strong willingness of the government to strictly supervise.

From the perspective of social co-governance, the social benefit value of e-commerce platforms under different response enthusiasm has a corresponding impact on the strategic choice of e-commerce platforms. Increasing the benefits of actively responding to infringement issues can effectively reduce the willingness of e-commerce platforms to passively respond to infringement issues.

#### Analysis of Rights Holders

For rights holders, the increase in the cost of active rights protection and passive rights protection will bias their strategic choices in the opposite direction. Therefore, the cost of active rights protection of rights holders should be reduced to increase their willingness to actively protect. When the rights holders are willing to actively protect their rights and interests, the higher the cost of docking between rights holders and the government or e-commerce platforms is, the more likely it is to cause rights holders to give up actively protecting their rights. Therefore, controlling the docking cost within a reasonable range will help enhance the stability of the rights holders' willingness to actively protect their rights and interests.

From the perspective of social co-governance, the increase in the loss value of active or passive rights protection will cause rights holders to change their strategic choice in the opposite direction. When the difference between the loss value of rights holders' active and passive rights protection is not large, it is difficult for rights holders to choose to actively protect their legitimate rights and interests; when the loss value increases during passive rights protection, rights holders are more willing to choose to actively protect their rights and interests. Therefore, the loss of rights holders' active rights protection should be reduced, and the sense of gains of rights holders' active rights protection should be improved. In other words, the loss of rights holders' active rights protection and the cost of docking with the platform should be reduced, and they should be controlled at a lower level than passive rights protection, which can better protect rights holders' active protection of their rights and interests.

#### Analysis of Stakeholders

The strategic choices of the government, e-commerce platforms, and rights holders are not only affected by the above factors but also changed due to the strategic choices of the other two stakeholders. When e-commerce platforms actively respond to infringement issues and rights holders are more likely to take the initiative to protect their rights and interests, the government will reduce its strict supervision rate, which is prone to lack of supervision; when the government strictly supervises and rights holders are more likely to actively protect their rights and interests, the probability will increase that e-commerce platforms will actively respond to infringement issues. Therefore, strict supervision activities by the government are essential, and rights holders should be encouraged to take the initiative to protect their rights and interests; when the probability of strict government supervision and the probability of e-commerce platforms actively responding to infringement issues are greater, rights holders will reduce their active protection rate and neglect to protect their related rights and interests.

### Policy Suggestions

Based on the above analysis, we put forward the following policy suggestions:

1. Control the costs of the government's strict supervision and encourage e-commerce platforms to actively respond to infringement issues; provide more diversified response methods for the docking of rights holders when defending their rights; urge e-commerce platforms to actively respond to infringement issues to eliminate docking costs; and ultimately, control costs within a reasonable limit.
2. Through the construction of a diversified policy system to minimize the cost of rights holders' active rights protection and clearly define the loss of rights holders' rights and interests to avoid infringement on the legitimate rights and interests of others due to mis-judgment or malicious rights protection, in the face of the losses that have already been incurred, it is necessary to stop the losses in time to increase the sense of gain of rights holders to actively protect their rights and interests.
3. We should reasonably adjust the penalties imposed by government supervisory agencies on e-commerce platforms and platform operators, give the government sufficient supervisory incentives, and increase the government's willingness to strictly supervise to sufficiently deter e-commerce platforms and platform operators. For e-commerce platforms that actively respond to infringement issues, the government should give rewards that can effectively enhance the enthusiasm of e-commerce platforms to deal with infringement issues. However, the incentives should not cause the government to incur excessive losses, and it is necessary to prevent the government from reducing its willingness to strictly supervise. Regarding the infringements of intellectual property rights caused by ineffective supervision, the government supervisory agencies should be punished to motivate them to provide supervision.
4. We should further strengthen the construction of a social environment that respects intellectual property rights, boost society's overall awareness of intellectual property rights, promote the public's supervision of the government and parties involved in e-commerce activities, improve the social benefits of strict government supervision, boost confidence in the intellectual property protection of e-commerce platforms, and enhance the sense of real gains of active rights protection by rights holders.

## Conclusions

This paper introduces the theory of social co-governance in the protection of e-commerce intellectual property rights and constructs a three-party evolutionary game model among the government, e-commerce platforms and rights holders. It analyses the strategic stability of stakeholders and the influence of individual factors and factor combinations on the stability of the evolutionary system. This paper verifies the validity of the analysis conclusions through simulation analysis and analyses the game model from the perspective of the government, e-commerce platforms, rights holders and interactions of the three stakeholders. Finally, it provides a reference for government policy making.

This paper introduces a certain number of reference elements in the process of constructing the social co-governance model. Among them, it is difficult to quantify the changes in docking costs and social benefits. At the same time, the participation of consumers (Meng et al., 2021), industry associations (Chen and Wu, 2019), government (Lu and Huang, 2021) and other social entities and the influence of the game sequence have not been considered. Therefore, solving the problem of numerical quantification, introducing more social subjects to construct a more diverse social co-governance model, analyzing the construction of a social co-governance model of intellectual property protection in the whole process of e-commerce activities, and putting forward more constructive opinions and suggestions for the construction of the “big protection” system of e-commerce intellectual property rights will be our next research direction.

## Data Availability Statement

The original contributions presented in the study are included in the article/supplementary material, further inquiries can be directed to the corresponding author/s.

## Author Contributions

JL and LH carried out the experiment. JL and CX wrote the manuscript. CX and LH helped supervise the project. All authors contributed to the article and approved the submitted version.

## Funding

This work was supported by the Shanghai Innovation Center of Reverse Logistics and Supply Chain (Shanghai Polytechnic University) (No. A30DB212103-06) and Shanghai Financial Technology Center (No. 2021-JK01-C).

## Conflict of Interest

The authors declare that the research was conducted in the absence of any commercial or financial relationships that could be construed as a potential conflict of interest.

## Publisher's Note

All claims expressed in this article are solely those of the authors and do not necessarily represent those of their affiliated organizations, or those of the publisher, the editors and the reviewers. Any product that may be evaluated in this article, or claim that may be made by its manufacturer, is not guaranteed or endorsed by the publisher.
